# Comorbidities and Disease Severity as Risk Factors for Carbapenem-Resistant *Klebsiella pneumoniae* Colonization: Report of an Experience in an Internal Medicine Unit

**DOI:** 10.1371/journal.pone.0110001

**Published:** 2014-10-15

**Authors:** Antonio Nouvenne, Andrea Ticinesi, Fulvio Lauretani, Marcello Maggio, Giuseppe Lippi, Loredana Guida, Ilaria Morelli, Erminia Ridolo, Loris Borghi, Tiziana Meschi

**Affiliations:** 1 Internal Medicine and Critical Subacute Care Unit, Parma University Hospital, Parma, Italy; 2 Department of Clinical and Experimental Medicine, University of Parma, Parma, Italy; 3 Geriatrics Unit, Parma University Hospital, Parma, Italy; 4 Laboratory of Clinical Chemistry and Hematology, Parma University Hospital, Parma, Italy; The University of Tokyo, Japan

## Abstract

**Background:**

Carbapenem-resistant *Klebsiella pneumoniae* (CRKP) is an emerging multidrug-resistant nosocomial pathogen, spreading to hospitalized elderly patients. Risk factors in this setting are unclear. Our aims were to explore the contribution of multi-morbidity and disease severity in the onset of CRKP colonization/infection, and to describe changes in epidemiology after the institution of quarantine-ward managed by staff-cohorting.

**Methods and Findings:**

With a case-control design, we evaluated 133 CRKP-positive patients (75 M, 58 F; mean age 79±10 years) and a control group of 400 CRKP-negative subjects (179 M, 221 F; mean age 79±12 years) admitted to Internal Medicine and Critical Subacute Care Unit of Parma University Hospital, Italy, during a 10-month period. Information about comorbidity type and severity, expressed through Cumulative Illness Rating Scale-CIRS, was collected in each patient. During an overall 5-month period, CRKP-positive patients were managed in an isolation ward with staff cohorting. A contact-bed isolation approach was established in the other 5 months. The effects of these strategies were evaluated with a cross-sectional study design. CRKP-positive subjects had higher CIRS comorbidity index (12.0±3.6 vs 9.1±3.5, p<0.0001) and CIRS severity index (3.2±0.4 vs 2.9±0.5, p<0.0001), along with higher cardiovascular, respiratory, renal and neurological disease burden than control group. CIRS severity index was associated with a higher risk for CRKP-colonization (OR 13.3, 95%CI6.88–25.93), independent of comorbidities. Isolation ward activation was associated with decreased monthly incidence of CRKP-positivity (from 16.9% to 1.2% of all admissions) and infection (from 36.6% to 22.5% of all positive cases; p = 0.04 derived by Wilcoxon signed-rank test). Mortality rate did not differ between cases and controls (21.8% vs 15.2%, p = 0.08). The main limitations of this study are observational design and lack of data about prior antibiotic exposure.

**Conclusions:**

Comorbidities and disease severity are relevant risk factors for CRKP-colonization/infection in elderly frail patients. Sanitary measures may have contributed to limit epidemic spread and rate of infection also in internal medicine setting.

## Introduction

In the era of antibiotic resistance and multi-drug resistant bacteria, the emergence and spread of carbapenem-resistant *Klebsiella pneumoniae* (CRKP), also known as carbapenemase-producing *Klebsiella pneumoniae*, has rapidly become a major health concern for hospitalized patients in industrialized countries [1]. Carbapenemases are β-lactamases that can hydrolyze carbapenems. Their outbreak in clinical isolates of Gram negative bacteria has been appreciated since the late 1990 s, with an increasing number of carbapenem-affine types identified ever since [2]–[3]. *Klebsiella pneumoniae* is the most frequent bacterial species associated with production of high-affinity carbapenemases. Their genes typically reside on transferable plasmids and are conventionally known as KPC. The first in vivo isolation of a CRKP strain dates back to 2000 in an intensive care unit of North Carolina [4]. During the following years, CRKP has been responsible of a large number of nosocomial outbreaks in many hospitals of Northeastern United States, causing a large number of deaths due to septic shock [5]. CRKP had reached all developed countries worldwide by the end of the 2000 s. In Europe, CRKP has an heterogeneous distribution, with some countries such as Poland and Greece, where CRKP infection is currently considered as endemic, and others such as Sweden and Portugal, where only few sporadic cases have been identified [6]. The first Italian CRKP isolation was recorded in Florence in 2009 in a patient with complicated intra-abdominal infection [7]. Since then, CRKP has rapidly spread throughout the country. This explains why Italy, that was classified only a few years ago as a nation with sporadic isolations, has been recently upgraded to an endemic country [6], [8]. Moreover, recent national data showed that CRKP is more frequently isolated from patients outside intensive care units (ICU), often admitted to geriatric or internal medicine wards [9]–[11].

CRKP is generally transmitted by contact and primarily colonizes lower intestinal tract and inguinal or perineal skin, so that active microbiological screening is regarded as an effective measure to prevent the onset of infection [12]. The main risk factors for colonization or infection identified in literature are critical illness and chronic diseases such as respiratory failure, prior antibiotic therapy and prior hospitalization [13]–[15]. When the infection occurs, it is generally associated with a bloodstream bacteremia followed by quick development of septic shock. Blood culture isolation of CRKP is an independent predictor of death, and the overall mortality ranges from 41% to 80% despite the establishment of appropriate antibiotic therapy [16]–[18]. Therapeutic options are indeed very limited, and include aminoglycosides, tigecycline, colistin, fosfomycin or even carbapenems themselves, when the minimal inhibitory concentrations (MICs) are ≤4 mg/L [19]–[21].

Few studies have investigated the characteristics of CRKP outbreaks in populations of elderly frail hospitalized patients with multi-morbidity. However, the quick spread of this pathogen in general internal medicine and geriatric wards around the globe may suggest that chronic diseases could play a major role as risk factors.

## Aims

We carried out a case-control study to explore the contribution of multi-morbidity and disease severity, measured through literature-validated indexes, in the onset of CRKP colonization/infection in a population of hospitalized, elderly and frail patients. With a cross-sectional study design, we have then described the changes in CRKP epidemic trend and rate of infection occurred after the institution of special sanitary measures, namely quarantine ward with staff cohorting management.

## Setting and Methods

The University Hospital of Parma, Italy, is a 1218-bed tertiary referral facility with approximately 51300 admissions per year. From August 2011 to May 2012, it has been the scenario of a pandemic outbreak of CRKP colonization and infection. This phenomenon especially involved older patients admitted to general internal medicine units. In order to limit the diffusion of CRKP, Healthcare Hospital Direction arranged an immediate transfer of all patients with a CRKP positivity to Internal Medicine and Critical Subacute Care Unit. This unit, a 94-bed large internal medicine area, split in smaller wards and organized by intensity of care, is mainly dedicated to the care of elderly frail patients.

All CRKP-positive patients were managed by contact isolation precautions. They received antibiotic therapy only in the presence of clinical or laboratory signs of infection. Colonized patients with no signs of infection were not treated.

Following the recommendations issued by Italian Health Ministry and Emilia-Romagna Region Health Authority [22], all high-risk patients admitted to our unit and all patients with clinical signs of infection underwent an active microbiological surveillance program consisting of a weekly rectal swab for CRKP detection. A patient was considered at high risk of CRKP infection if he/she had been transferred from another hospital or from a community nursing home, hospitalized in the previous 60 days, transferred from an intensive care unit, in contact (i.e., in the same room) with a CRKP-positive patient or completely bedridden for at least 3 days. In case of clinical signs of infection, other microbiological tests, such as blood or urine culture, were prescribed whenever appropriate and according to the clinical characteristics of each patient. Surveillance was continued until the patient was discharged or had 3 consecutive rectal swabs negative for CRKP detection.

Moreover, given the high number of CRKP-positive patients, from October 2011 (two months after the beginning of the outbreak) to February 2012, under the indication of Healthcare Hospital Direction, a 14-bed isolation ward with a staff-cohorting management was activated. This ward was reserved only to CRKP-positive patients with dedicated health care professionals that could not come to contact with other CRKP-negative patients. A simple model validated in the literature and already used to control multi-drug resistant infection outbreaks was used [23]–[24]. This 14-bed isolation ward was closed at the end of February 2012, since its maintenance was considered no longer cost-effective by Healthcare Hospital Direction, due to the rapid and consistent decrease of new cases of CRKP positivity. Colonized or infected patients admitted thereafter were only managed by contact isolation precautions until the end of the observation period. Active microbiological surveillance was continued throughout the study period, regardless of the management by staff cohorting or contact isolation.

**Figure 1:**
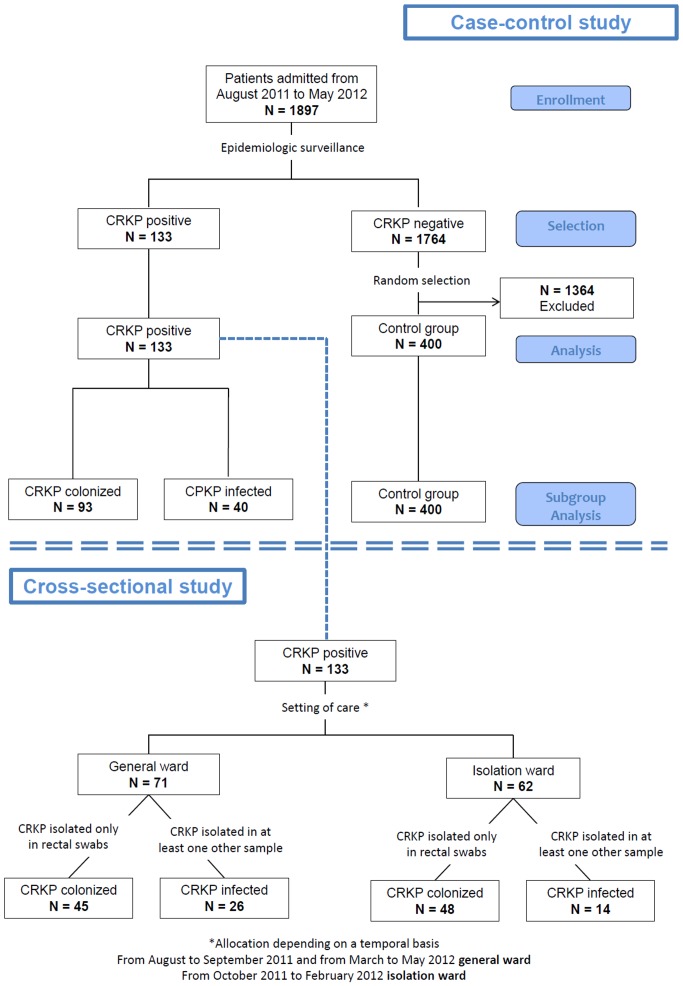
Summary of the study design.

To assess whether comorbidity number and severity is a risk factor for CRKP colonization/infection, we carried out a case-control study (Figure 1). We reviewed all clinical records of patients admitted to our unit from August 2011 to May 2012 (1897 subjects) to check CRKP status. CRKP positivity was defined as the presence of at least one biological sample positive for CRKP. All patients admitted to our unit who failed to meet the requirements for epidemiological surveillance program or who had microbiological analysis (i.e., all rectal swabs) negative for CRKP were considered as CRKP-negative. For the study purposes, all consecutive CRKP-positive patients (133 subjects) who were identified during the study period were considered as cases. We also randomly selected 400 clinical records of CRKP-negative patients and considered them as controls.

Moreover, subjects in whom CRKP was isolated only in rectal swabs performed for epidemiological surveillance reasons and with other biological samples resulted negative for CRKP, were considered as CRKP-colonized. Subjects who had clinical signs of infection and at least one biological sample, other than rectal swab, positive for CRKP were considered as CRKP-infected, irrespective of their rectal swab status.

In both cases and controls, a well-trained physician recorded age, primary diagnosis, type and severity of comorbidities according to the Cumulative Illness Rating Scale (CIRS) score [25], overall hospital length of stay, biological sample of CRKP isolation and genotypic characteristics when available, possible clinical signs of CRKP infection and final outcome (discharge or death).

To describe changes in epidemiologic trend after the institution of the staff cohorting quarantine ward, we also carried out a cross-sectional study (Figure 1). We considered only CRKP-positive patients, classified as colonized or infected according to the above criteria. Incidence of CRKP-positivity was calculated as the number of newly diagnosed cases per month related to the overall number of admissions in the same month. Outbreak control was defined as a persistent decline or stability in the trend of monthly incidence. The rate of infection was calculated as the percentage of CRKP-infected patients related to the overall number of CRKP-positive patients. Data about CRKP genotype was also recorded for each patient whenever available.

The protocol was authorized by the Ethics Committee of Healthcare Hospital Direction of Parma University Hospital, that is also supported by a Scientific Research Board and a specific Research Plan approved by Geriatric-Rehabilitation Department. All patients signed a specific informed consent form at time of admission, stating that the patient authorizes personal and clinical data treatment and analysis in aggregate way for scientific-statistic research purposes according to Italian legislation. All the clinical investigations were performed according to the principles expressed in the Declaration of Helsinki.

For descriptive purposes, baseline characteristics of cases and controls were compared using a χ^2^ test (Mantel-Haenszel method) and ANOVA model for categorical and continuous variables, respectively.

Bivariate tests were used: χ^2^ for dichotomic variables was used to test the significance of categorical covariates. Parametric (ANOVA) and non-parametric test, the Wilcoxon signed-rank test, were used to assess the significance of continuous covariates. Logistic regression analysis was used to examine the relationship between CIRS category in the case group compared to control group.

All analyses were performed using SAS (version 8.2, SAS Institute, Inc., Cary, NC) with a statistical significance level set at p<0.05.

## Results

The total number of CRKP-positive patients observed from August 2011 to May 2012 (42 weeks) was 133 (75 M, 58 F; mean age 79±12 years). The control group (400 patients) included 179 males and 221 females, with a mean age of 79±10 years. In the same period, the overall number of patients admitted to our unit was 1897, so that the overall incidence of CRKP positivity was 7%.

As also shown in Figure 1, 93 patients out of 133 cases (70%), were classified as simply CRKP-colonized, while 40 patients (30%) were CRKP-infected. The biological samples, other than rectal swabs, in which CRKP was first isolated in infected subjects were blood or vascular catheters in 21 cases (52.5%), urine in 13 cases (32.5%), phlegm in 3 cases (7.5%) and surgical wound swabs in other 3 cases (7.5%).

CRKP-positive patients had a large number of comorbidities with a high degree of clinical complexity, as attested by high values of CIRS comorbidity and CIRS severity indexes (Table 1). Both indexes were significantly higher in CRKP-positive patients than in controls (comorbidity index 12.0±3.6 vs 9.1±3.5; p<0.0001; severity index 3.2±0.4 vs 2.9±0.5; p<0.0001) (Table 1). The comparison of the mean values of each CIRS score item between cases and controls is shown in Table 2. CRKP-positive patients had a significantly higher burden of cardiovascular, respiratory, renal, neurological and musculoskeletal disorders than CRKP-negative patients. Cardiovascular and respiratory diseases were indeed the most frequent among the case group, with a prevalence of 64% and 54%, respectively. The role of cardiovascular, respiratory, neurological and renal diseases as risk factors for CRKP colonization was confirmed by logistic regression analysis, and appeared to be independent of age, sex, head and neck, upper and lower gastrointestinal, hepatic, urological, endocrine and psycho-behavioral diseases (Table 3). Moreover, a high CIRS severity index was found to be the leading risk factor for CRKP positivity (odds ratio 13.3; 95% CI 6.88–25.93; p<0.0001).

**Table 1:** Characteristics of the study population.

	CASES n = 133	CONTROLS n = 400	p*
Age (years) (mean ± SD)	79±12	79±10	0.50
Men (n, %)	75 (56.4)	221 (55.3)	0.94
CIRS comorbidity Score**	**12.0±3.6**	**9.1±3.5**	**<0.0001**
CIRS severity Index***	**3.2±0.4**	**2.9±0.5**	**<0.0001**
Number of comorbidities****	**3.8±1.2**	**3.3±1.5**	**<0.0001**
Hospital length of stay (days)	**35±24**	**18±12**	**<0.0001**

* Age- and sex-adjusted (where possible).

** CIRS comorbidity Score was calculated as the sum of each of the first 13 items of organ or system disease, excluding only psycho-behavioral disease item. For each item, a score ranging from 0 to 4 can be given. 0 means absence of disease, while 4 means a potential life-threatening disease.

*** CIRS severity Index represents the number of times that a patient ranks 3 or 4 points in each of the 14 items of CIRS (psycho-behavioral disease included).

**** Number of comorbidities was calculated as the number of acute or chronic illnesses that were recorded during the hospital stay for each patient.

CIRS  =  Cumulative Index Rating Scale.

**Table 2:** Differences between singular CIRS categories in the cases compared to controls.

CIRS CATEGORY*	CASES n = 133	CONTROLS n = 400	p**
Heart disease	**2.12±1.52**	**1.13±1.42**	**<0.0001**
Hypertension	**1.34±1.12**	**0.73±0.91**	**<0.0001**
Vascular, hematological disease	0.84±1.55	0.67±1.31	0.20
Respiratory disease	**1.97±1.78**	**1.05±1.54**	**<0.0001**
Eye, ear, nose, and throat disease	0.12±0.68	0.10±0.43	0.73
Upper gastrointestinal disease	0.30±1.02	0.37±0.99	0.53
Lower gastrointestinal disease	0.47±1.23	0.50±1.22	0.76
Liver disease	0.12±0.67	0.15±0.62	0.62
Kidney disease	**1.03±1.55**	**0.61±1.23**	**0.001**
Other genitourinary disease	0.14±0.71	0.21±0.74	0.28
Musculoskeletal, skin disease	**0.27±1.00**	**0.58±1.23**	**0.009**
Neurological disease	**1.30±1.72**	**0.96±1.51**	**0.03**
Endocrine, metabolic disease	0.72±1.20	0.77±1.44	0.70
Psychiatric or cognitive disease	1.30±1.81	1.29±1.53	0.73

* All the single CIRS items are listed in this Table. For each item, a score ranging from 0 to 4 can be given. 0 means absence of disease, while 4 means a potential life-threatening disease.

** Age- and sex-adjusted.

CIRS  =  Cumulative Index Rating Scale.

**Table 3:** Odds of association between CIRS category in the case group (n = 133) compared to control group (n = 400).

	ODDS RATIO	95% CI	p*
**CIRS severity Index**	**13.3**	**6.88**–**25.93**	**<0.0001**
Hypertension	1.96	1.43–2.70	<0.0001
Heart disease	1.68	1.36–2.09	<0.0001
Respiratory disease	1.46	1.25–1.70	<0.0001
Vascular, hematological disease	1.39	1.16–1.66	0.0004
Kidney disease	1.37	1.14–1.64	<0.0001
Neurological disease	1.33	1.12–1.57	0.001

* Also adjusted for age; sex; eye, ear, nose and throat disease; upper and lower gastrointestinal disease; liver disease; other genitourinary diseases; endocrine and metabolic disease; psychiatric and cognitive diseases.

CIRS  =  Cumulative Index Rating Scale.

A subgroup analysis performed on infected vs colonized patients showed that neither the CIRS comorbidity score (11.5±3.1 vs 12.2±3.8, p = 0.36), nor the CIRS severity index (3.1±0.3 vs 3.2±0.4, p = 0.55) were statistically different between these two groups.

Mean hospital length of stay was significantly longer in CRKP-positive patients than in the control group (35±24 vs 18±12 days, p<0.001), as also shown in Table 1.

Twenty-nine CRKP-positive patients out of 133 died during hospitalization (21.8%). However, the mortality rate was not significantly different compared to that observed in the control group (61/400 subjects, 15%; p = 0.08). When only CRKP-infected patients were considered, the hospital mortality rate was 47.5%, while it was much lower in CRKP-colonized patients (10 patients out of 93; 10.7%).

**Figure 2:**
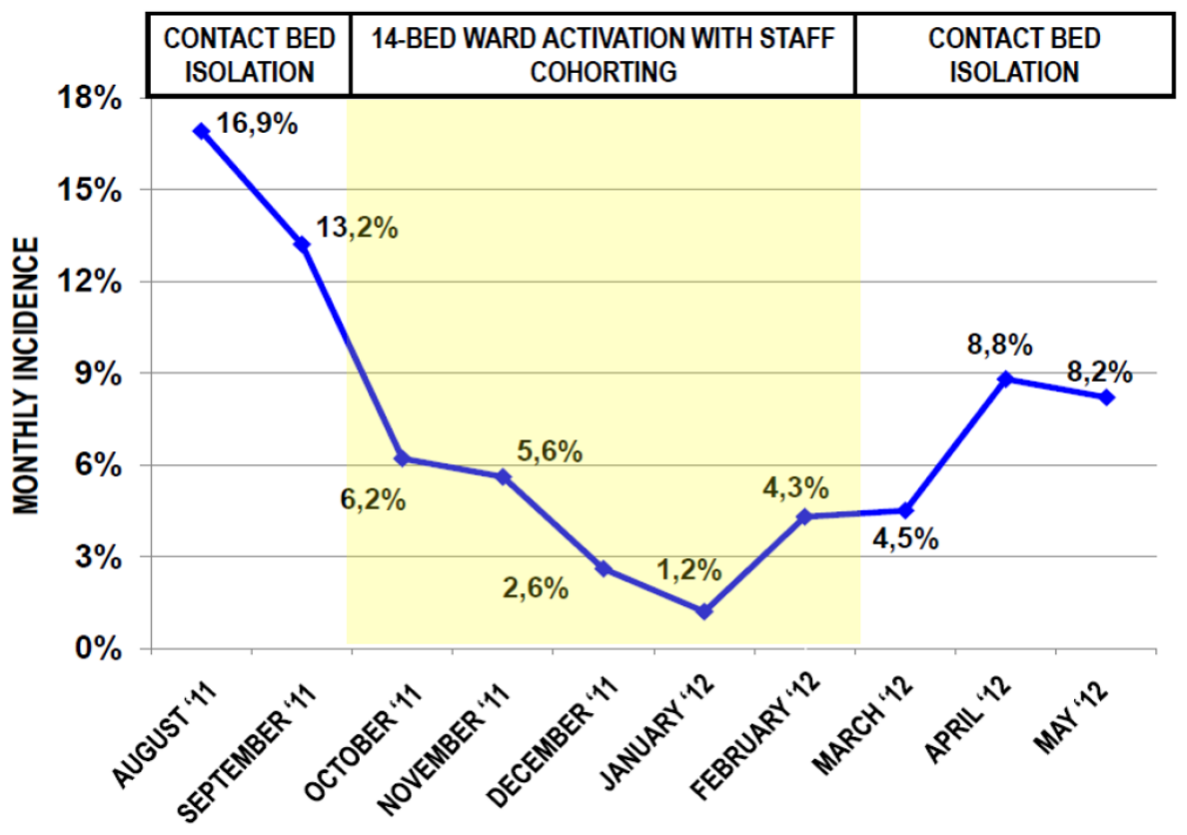
Trend in monthly incidence of CRKP positive cases in the period studied. (CRKP  =  Carbapenemase-resistant *Klebsiella pneumoniae*).

**Figure 3:**
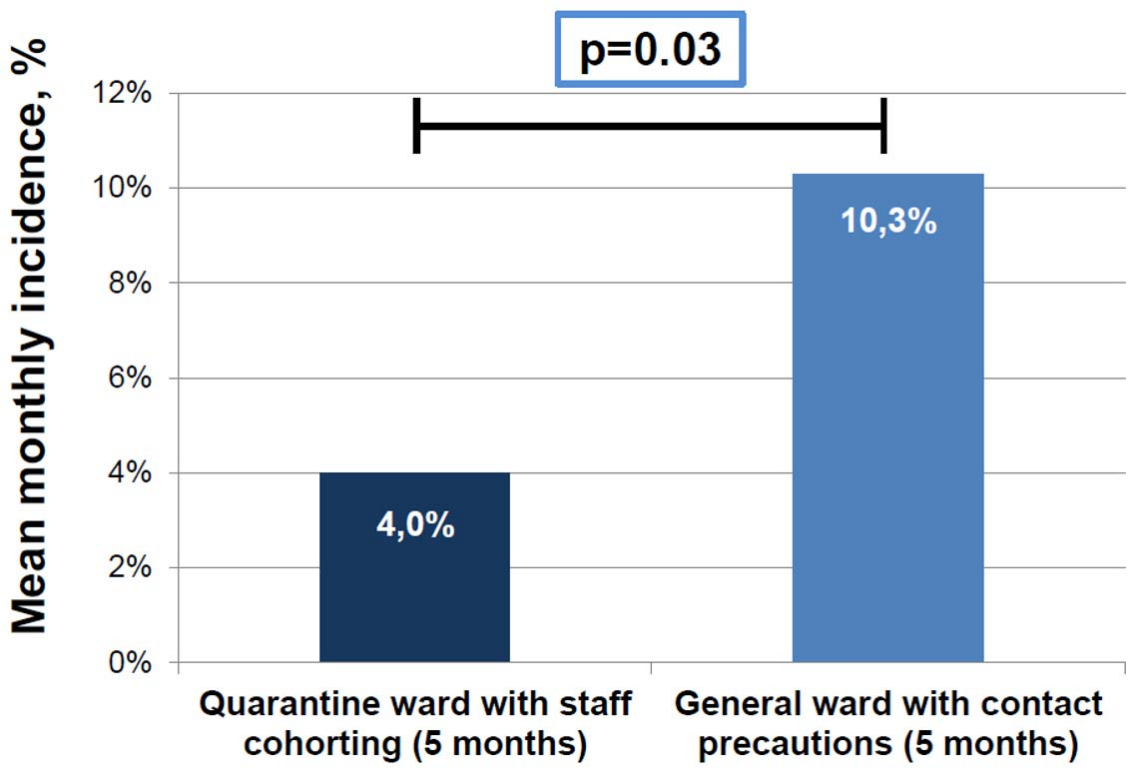
Comparison of mean monthly incidence of new cases of CRKP positivity between quarantine ward management period and general ward management period. Statistical analysis performed with analysis of variance. (CRKP  =  Carbapenemase-resistant *Klebsiella pneumoniae*).

A total number of 71 CRKP-positive patients was managed in the general medical ward with simple contact bed isolation precautions (from August to September 2011 and from March to May 2012). Sixty-two CRKP-positive patients were managed with staff cohorting approach in the isolation ward (from October 2011 to February 2012), as shown in Figure 1. The highest monthly incidence was observed in the first two months of the epidemic outbreak (23 and 18 new cases per month, with a 16.9% and a 13.2% monthly incidence, respectively). After the activation of the staff cohorting isolation ward, a net decrease in incidence of new cases was observed, with an average of 8 cases per month (range 3–13). A statistical analysis performed with Wilcoxon signed-rank test demonstrated that the decrease between the first (August-September 2011) and the second period (October 2011–February 2012) was statistically significant (p = 0.04). After closure of the quarantine ward at the end of February 2012, an increased incidence of new CRKP cases was recorded, although not statistically significant (second period October 2011–February 2012 vs third period March–May 2012, p = 0.08). The overall monthly incidence trend is shown in Figure 2. The mean monthly incidence during the period of quarantine ward management was 4.0%, whereas it was 10.3% in the period of general ward management. This difference was statistically significant (p = 0.03), as also shown in Figure 3.

The rate of CRKP-infected patients was similar in the subgroup managed by contact isolation approach in general ward (26 subjects out of 71, 36.6%) and in the subgroup managed by staff cohorting approach in isolation ward (14 subjects out of 62, 22.5%, p = 0.07 with Mantel-Haenszel chi-square). Mortality was also not statistically different in the two groups (16.1% in quarantine ward vs 26.7% in general ward, p = 0.14 with Mantel-Haenszel chi-square).

Information about the CRKP genotype was available in 102 out of 133 patients. In 76 patients (74%) the strain was positive for blaKPC or other type A carbapenemases, in 12 patients (12%) the strain was positive for type B carbapenemases, namely New Delhi metallo-beta-lactamase (NDM-1), whereas in the remaining 14 patients (14%) the isolated CRKP strain was genotypically classified as not carbapenemase producer. The infection and mortality rates were higher in patients positive for NDM-1-producing strains (50% and 42%, respectively) than in patients positive for type A carbapenemase-producing strains (25% and 17%, respectively) and in patients positive for non-carbapenemase-producing strains (29% and 7%, respectively). The groups exhibited also similar CIRS comorbidity score and severity index.

## Discussion

CRKP has rapidly emerged as a notable cause of nosocomial infections in Italy, with a high potential for developing large pandemic outbreaks [9], [11], [18], [26]–[28]. In this study, we have demonstrated that chronic comorbidities, namely cardiovascular, respiratory, renal and neurological impairments, along with disease severity, play a relevant role as risk factors for CRKP colonization/infection in elderly hospitalized subjects. To our knowledge, this is the first study in a population of frail elderly with a large number of comorbidities admitted to an internal medicine setting. The present investigation is also one of the few that considered this health issue from a genuine clinical perspective, more focused on disease-related risk factors for CRKP colonization/infection rather than on microbiological or molecular issues. The main limitations are the retrospective design, the lack of data about prior antibiotic exposure and functional status of patients before admission. Moreover, comorbidities were assessed through CIRS, which is not completely objective even when performed by a trained physician, although well-validated in medical literature.

Some risk factors for CRKP colonization and infection have already been investigated in the literature. For example, the importance of prior antibiotic exposure, especially to carbapenems, has been earlier emphasized [13]–[15].

Few studies have assessed the role of specific comorbidities as risk factors for developing a CRKP colonization or infection. There is actually only one study, performed in an ICU setting, that has associated a specific chronic disease, namely chronic obstructive pulmonary disease (COPD), with the risk of CRKP positivity [14]. Our data seem to confirm this finding, since CRKP-positive patients do have a higher degree of respiratory impairment than control subjects, although we also showed that cardiovascular, neurological and kidney disease may represent other substantial risk factors. Therefore, in our experience, a high number of comorbidities is an outstanding element influencing the risk of becoming CRKP-positive (Tables 2–3).

As also shown in an ICU context [13]–[16], disease severity is another relevant risk factor, irrespective of the number of comorbidities. According to our data, patients with a high CIRS severity index actually have an impressive 13-fold risk of becoming CRKP-positive, regardless of single organs or systems involved in disease. Thus, we can argue that CRKP, both in ICUs and in internal medicine wards, mainly affects frail complex patients with severe prognosis. In this subset of patients, in whom the clinical course is often difficult to manage, CRKP provides a new, sometimes fatal, element of clinical complexity. However, it is also noteworthy that a higher risk for infection is a finding common to resistant organisms in these patients. CRKP has actually epidemiologic features similar to the ones of other emerging nosocomial pathogens.

Therefore, it should be no longer only considered a typical ICU concern, but also a pathogen that internal medicine health care professionals and physicians need to deal with.

However, despite the high comorbidity burden of our patients, the recorded mortality rate was surprisingly not as high as that reported in literature [16]–[18]. As a matter of fact, the number of deaths was not significantly different in all CRKP-positive patients than those recorded in the control group of CRKP-free patients. However, when the CRKP infection occurs, the mortality rises due to the high risk of fatal septic shock. The mortality rate that we have recorded in CRKP-infected subjects is actually very similar to that previously reported in our country by Tumbarello and colleagues in a multicenter study [18]. We can hypothesize that simple rectal colonization by CRKP does not significantly modify the clinical course of frail elderly patients with multiple comorbidities. However, when the infection occurs, it significantly threatens survival of these patients, similarly to what has been shown in ICU patients. Further research is needed to better understand factors that induce the transformation of colonization into infection, both in intensive care and internal or geriatric medicine settings.

In our experience, we have also observed the effects of an isolation ward activation with a staff cohorting management, as an attempt to limit the contacts of CRKP-negative patients with colonized or infected patients. However, the cross-sectional design of the study does not allow to establish whether or not the variations in incidence of CRKP positivity are causally related to different management strategies. Moreover, patients were managed by staff cohorting or by contact bed precautions on a temporal basis and not on a random assignment. However, the remarkable decrease of monthly incidence observed after the institution of these measures, combined with the new increase detected after the isolation ward was closed (Figure 2), indirectly suggests that this approach may be related to a decrease in the incidence of new cases. Our data also indicate that this strategy may help reducing the rate of CRKP-positive patients that develop a clinical infection by CRKP. Further research is needed to confirm these hypotheses. Staff cohorting management is a simple sanitary measure, which was proven effective in other nosocomial pandemics [24]–[25]. Some recent studies in Greece demonstrated that the implementation of basic hand hygiene and contact precautions by health care professionals, isolation of CRKP-positive patients in dedicated rooms and staff cohorting are effective, either alone or in combination, to limit the spread of CRKP, although in surgical and intensive care settings [29]–[30]. There are also reports in which even more strict hygienic measures have been applied, such as daily chlorhexidine baths for positive patients, environmental surveillance for CRKP detection on surfaces at high risk for contamination and meticulous daily environmental cleaning [31]–[32]. These measures are obviously more difficult to be established in a non-ICU setting. Nevertheless, it is plausible that the higher number of hygienical precautions are established, the more effective is the prevention protocol for limiting multi-drug resistant outbreaks, even in geriatric frail patients.

However, possible biases should also be taken into account for the interpretation of our results. For example, considering that the lowest incidence in our experience was reported during winter months, we cannot exclude that CRKP colonization/infection outbreaks exhibit a seasonal periodicity. Our data are in agreement with those from a large multicenter epidemiologic study that some years ago evaluated non-multidrug-resistant *Klebsiella pneumoniae* strains [33]. Interestingly, opposite data emerged from a subsequent study conducted in a temperate climate area of the United States [34].

## Conclusions

CRKP outbreaks are becoming a relevant health issue, not only in an intensive care setting, but also in internal medicine and geriatric wards. Special sanitary measures, such as epidemiologic surveillance with weekly rectal swabs, bed isolation and quarantine ward activation with staff cohorting management may play a role in controlling the epidemic spread.

Our experience clearly suggests that a high number of comorbidities, and particularly cardiovascular, respiratory, renal and neurological disease, and high disease severity represent relevant risk factors for colonization and infection in elderly frail patients. Therefore these data open new scenarios for the most suitable strategies for issuing effective preventive measures in those patients at higher risk, not only admitted in ICU setting but also in general internal medicine wards. The clinicians' awareness and knowledge of this problem will therefore be crucial in the future for management of these severe hospital pandemics. Future research is needed to assess the relationships between the domains of frailty and chronic illness and the risk for CRKP colonization or infection.
